# Community perceptions of behaviour change communication interventions of the maternal neonatal and child health programme in rural Bangladesh: an exploratory study

**DOI:** 10.1186/s12913-016-1632-y

**Published:** 2016-08-16

**Authors:** Atiya Rahman, Margaret Leppard, Sarawat Rashid, Nauruj Jahan, Hashima E. Nasreen

**Affiliations:** 1 Research and Evaluation Division, BRAC, BRAC Centre, 75 Mohakhali, Dhaka, 1212 Bangladesh; 2 University of Aberdeen, Aberdeen, Scotland; 3 BRAC Health Population and Nutrition Programme, BRAC, 75 Mohakhali, Dhaka, 1212 Bangladesh

**Keywords:** Behaviour change, BRAC, Communication, Community-based intervention, MNCH, Rural Bangladesh

## Abstract

**Background:**

This qualitative study explored community perceptions of the components of the behaviour change communication (BCC) intervention of the BRAC Improving Maternal, Neonatal and Child Survival (IMNCS) programme in rural Bangladesh.

**Methods:**

Semi-structured interviews, key informant interviews, focus group discussions and informal group discussions were conducted to elicit community views on interpersonal communication (IPC), printed materials, entertainment education (EE) and mass media, specifically (a) acceptance of and challenges presented by different forms of media, (b) comprehensibility of terms; printed materials and entertainment education and (c) reported influence of BCC messages.

**Results:**

IMNCS BCC interventions are well accepted by the community people. IPC is considered an essential aspect of everyday life and community members appreciate personal interaction with the BRAC community health workers. Printed materials assisted in comprehension and memorization of messages particularly when explained by community health workers (CHW) during IPC. Enactment of maternal, neonatal and child health (MNCH) narratives and traditional musical performances in EE helped to give deep insight into life’s challenges and the decision making that is inherent in pregnancy, childbirth and childcare. EE also improved memorization of the messages. Some limitations were identified in design of illustrations which hampered message comprehension. Some respondents were unable to differentiate between pregnancy, delivery and postpartum danger signs. Furthermore some women were afraid to view the illustrations of danger signs as they believed seeing that might be associated with the development of these complications in their own lives. Despite these barriers, participants stated that the IMNCS BCC interventions had influenced them to take health promoting decisions and seek MNCH services.

**Conclusions:**

Community based maternal and newborn programmes should revise BCC interventions to strengthen IPC, using rigorously tested print materials as aids and stand-alone media. Messages about birth preparedness (especially savings), recognition of danger signs and immediate self-referral to biomedical health services should be carefully aligned and effectively delivered to women, men and older members of the community. Messaging should utilize gendered storyline and address the seasonal cycles of conception, birth, antenatal, post-natal care and childhood illnesses. Future research should identify how best to combine IPC, printed materials, traditional cultural forms, and incorporate use of social media and mass media in different field situations.

**Electronic supplementary material:**

The online version of this article (doi:10.1186/s12913-016-1632-y) contains supplementary material, which is available to authorized users.

## Background

Maternal, neonatal and child health (MNCH) programmes are of high national importance in Bangladesh [1–5]. Theoretically, by using communication channels to promote healthful behaviours and by creating a supportive environment, individuals will be able to consistently engage in health-promoting behaviours [6]. In Bangladesh a large number of MNCH programmes have been initiated, targeting both rural and urban poor populations [1, 4, 7, 8]. BCC strategies are considered to be an integral part of these services. These strategies range from individual face-to-face contacts to the use of traditional media like folk songs and street theatre [1, 7]. BCC has been primarily women-focussed in Bangladesh and other countries [9]. However, more recently men have been recognised as important partners in sexual and reproductive health and in child care [10, 11]. Long-term behavioural change among marginalized groups is limited as a result of education only [12] whereas health communication through a variety of media has been found to be effective in both increasing knowledge and facilitating behavioural change [13, 14]. Reaching the marginalised poor with a carefully designed programme to change health related behaviour is considered essential for any effective health system [15]. Although at lower levels of literacy women are more functionally literate than men in both urban and rural areas [16] with limited access to any form of media their knowledge on MNCH can be enhanced [14]. However, because women’s roles are defined by the family and community network, community-based intervention is designed to mobilize both women and men, across generations so that they can reinforce each other’s new behaviour [17]. Many organizations employ BCC materials in maternal and child health programme. However pilot testing of these materials and evaluation and analysis to determine their relevance and effectiveness is limited [1, 7, 18–20].

Improving maternal neonatal and child survival (IMNCS) programme of BRAC was initiated to reduce maternal, neonatal and child mortality and morbidity in poor communities [4, 21]. It started in Nilphamari district in northern Bangladesh and was gradually expanded to three other districts in 2008 and a further six in 2010. While the BRAC IMNCS programme mainly targets pregnant women, mothers of newborns and under-5 children, it also includes family members and influential community people in its target population. Community health workers (CHW) namely *Shasthya Shebika* (SS) and *Shasthya Karmi* (SKs) are the core providers of the programme. They are responsible for delivering services at grass root level [4, 21]. SSs, the frontline CHWs, are selected from the community having primary level education. They should be married and aged between 25 and 40 years. After recruitment, SS receive basic training from programme organizer (PO) and managers on family planning, pregnancy identification and conduct of antenatal visit with SKs; assistance at delivery, conduct of postnatal visit, and care of newborns and under five children. SK is the second level CHW. They are also the residents of the locality, aged between 20 and 35 years and have higher secondary level education. Like the SS, after joining the programme, SKs receive training on pregnancy identification, antenatal care (ANC), identification of maternal complication, postnatal care (PNC), essential newborn care and under five child care services. In addition, SK are trained to treat ten common illnesses; identify tuberculosis (TB) patients and provide directly observed treatment, short-course (DOTS); and help to prepare report monthly. Usually an SK supervises 10 SSs. Both the SS and SKs attend regular refresher training. To bring about behaviour change in maternal and child health practices at the community level, the IMNCS programme introduced BCC interventions from its initiation in 2006. The purpose of this study was to explore community perceptions of BCC interventions of the BRAC IMNCS programme in rural Bangladesh.

### BCC intervention in IMNCS programme

Both SS and SK provide information and support for behaviour change through face to face counselling and group discussion with family members. The interpersonal communication between SK, the pregnant woman and her family members is buttressed by showing illustrations printed on flipcharts. Messages delivered by CHWs include: pregnancy care, birth preparedness, safe delivery, postpartum care, neonatal and child health and include topics such as: nutrition, safety, rest taking and cleanliness during pregnancy, the need for antenatal check-up. Importantly, the danger signs of pregnancy, delivery and the postpartum period are discussed.

Four different types of posters and two stickers were distributed to the women as part of the BCC intervention. Posters illustrate first, a healthy mother and child with the message “*if you find any problem in pregnancy, do not delay. We want healthy mothers, healthy children, and healthy neonates*”; second, the eight newborn danger signs with the images of newborns who are unable to suck breast milk, have fever, cold, continuous vomiting, chest in-drawing, skin pustules, umbilical infection, convulsion and lethargy; third, a poster on ‘signs of pneumonia and its management’ and fourth, ‘management of diarrhoea’ with images showing the relevant signs, remedies and management. One sticker depicts the maternal danger signs of pregnancy, delivery, and post delivery period’. The danger signs include high fever; severe headache and blurring of vision; prolonged labor; convulsion; and hemorrhage or excessive bleeding. The second sticker is a smaller version of the poster illustrating neonatal danger signs. Both stickers and the poster on children’s danger signs also highlight procedures for accessing health facilities.

Posters and stickers are posted on the wall of the woman’s house by the CHWs to reinforce knowledge and awareness. The PO’s mobile phone numbers are listed at the bottom of each poster and sticker so that they can be contacted in case of emergencies.

The mass media approach to MNCH BCC includes folk songs (locally termed as *jaarigan*) and street theatre (*naatak*). The programme generally hires a local team to organize and perform the events according to prepared script. The topics to be addressed by folk songs and street theatre are selected by the programme personnel. The folk song and street theatre initiative deliver messages specifically on antenatal care, safe delivery, postpartum care, family planning, infant and child health. The street theatre also introduces the role of SS and SK and demonstrates how the practice of traditional beliefs can negatively affect pregnancy outcomes.

## Methods

### Design

The BCC component of the IMNCS programme was designed to use the coherent multi-dimensional approach described above. The messages that CHWs learn during training and refresher courses are delivered through IPC, printed materials and EE. The periodic evaluations of the BRAC IMNCS programme reveal macro level changes in service use and knowledge of community members about MNCH promoting behaviours [4, 22–24]. The aim of this study was not therefore to assess the technical quality of the messages, nor extent of behaviour change in the community. This qualitative study was designed by the BRAC Research and Evaluation Division (RED) led by Dr Hashima E Nasreen, in consultation with Dr Leppard, a social anthropologist/nurse/midwife with experience of health systems, health communication and a long term professional resident in Bangladesh and experienced RED researchers to explore the community perceptions of the multidimensional BRAC IMNCS BCC interventions in order to inform the next stage of programming. Our research questions therefore sought to explore the extent to which each of the BCC tools were acceptable and comprehensible to the community members, specifically: (a) Community members acceptance of and challenges presented by different forms of media, (b) Community members comprehension of terms used, illustrated printed materials and education entertainment and (c) reports of the influence of BCC messages. Our interview and discussion guides included topics such as

- Awareness of BCC media and messages
- To what extent are BCC media attractive to the community members?
- Do community members consider the source of messages credible? Why? Why not?
- Does the BCC component reach the heart (emotions) and head (knowledge and understanding) of the community members?
- Does the BCC component create trust? In whom? Call for action? By whom?
- Are these messages able to influence community practice?
- Do the messages appeal to the beliefs of the target audience?

We used different types of data collection methods in order gather more comprehensive data, that increases validity, enhances understanding of the studied phenomenon and reduces the possibility of non-sampling error [25]. In this study the within-method triangulation process was followed [26]. Thus semi-structured interviews, key informant interviews (KII), focus group discussions (FGD) and informal group discussions (IGD) (Table 1) were used (See also Additional files 1 and 2). Semi-structured interviews were used to gather data pertinent to our research questions, giving respondents the possibility to elaborate their answers and researchers to do some probing. KIIs enabled us to probe more deeply. FGDs were useful because they spark the participants’ sharing of narratives and discussion about a range of experiences and opinions related to the research topics in a relative short space of time. Like interviews, FGD data consists of verbal reports (compared with direct observation methods), in all groups there was the risk that perceived social status of other participants and researchers could constrain participants input. However, in Bangladesh FGDs, skilfully led by researchers who are often perceived as educated, high status persons who are unusually, in the eyes of the discussants, willing to sit with them and ‘listen to our words’ and ‘take our words to Dhaka’ yield rich data in a context where local status differentials among the discussants are smoothed. BRAC’s RED researchers and research assistants (RAs) are trained, skilled and experienced in conducting FGDs. It is relatively easy with little preparation to conduct FGDs in Bangladeshi homes. Mats made of reeds or woven plastic sacking are laid out on ‘*verandahs*’ or in the courtyards of rural homes and the FGD proceeds. Each one of our focus groups was conducted in a different home. For each category of respondents one FGD was conducted in each of the two unions selected for the research.

**Table 1:** Types of interviews by respondents categories

Type of respondents	Unit of interview			
	Semi-structured interview	KII^a^	IGD^a^	FGD^a^
(1)Pregnant women	8	3	-	2
(2)Mothers of neonates and under 5 year old children	15	3	-	2
(3)Mother in laws of neonates and under 5 year old children	15	3	-	2
(4)Fathers of neonates/children under 5 years and the husbands of pregnant women	12	3	-	2
(5)CHWs			3	
Total	50	12	3	8

^**a**^Note: *KII* key informant interview, *FGD* focus group discussion, *IGD* informal group discussion

### Study area and population

The study was conducted in two unions of Nilphamari sadar upazilla (Districts are divided into sub-districts called upazilla. Each upazilla is sub-divided into unions) in March and April, 2010. The population of interest was the targeted population described in Table 1. All the pregnant women and their details (home address, husband’s name, date’s of ANC, delivery and PNC, record of maternal complications if any, place of delivery, name of SS and SK as caregiver) are recorded in the programme register. From this register, a list was purposively prepared of currently pregnant women and of (previously pregnant) mothers of neonates and under 5 years children from this register. These women were approached in person during a rapport building phase and if they gave consent were later interviewed along with their respective their respective mothers-in law and husbands.

In this way we were able to meet numbers of community members considered adequate for this type of qualitative research to ensure validity [27, 28]. Indeed Maxwell in his discussion of the use of numbers in qualitative research cautions that ‘precision is not the same as validity’ [29]. For our study it was important that the data were both reliable and valid to yield inform the next stage of detailed programme planning. Questions of generalisability and replicability have already been addressed in the quantitative surveys [21]. Of the possible participants those living at periphery were omitted because of the limited human and financial resources available for the study, and time required for researchers to reach them. We preferred therefore to use our time resource to gather rich data in more accessible areas. Time ‘in the field’ is a recently proposed criterion for good qualitative research [30, 31] that is aligned to the notion of data saturation. In our case, while we are aware of the challenges that the programme faces in reaching remote populations, itself a physical (time, distance and transport) communication issue, we considered that the exploratory study conducted among a more accessible population would yield adequate information about the existing BCC for programme planning and development purposes.

### Data collection strategy

Four female persons have been deployed as RAs. They were graduates from Sociology and Anthropology with basic training in qualitative methodology and 2–3 years’ experience in qualitative data collection procedure. They were responsible for conducting semi-structured interviews, IGD and FGDs. KIIs were done by the Bangladeshi authors. In the field notes were taken by the RAs during interviews which were also recorded on tape. After each interview, a verbatim transcription was made by listening to the tapes and field notes were made in Bengali. These were later translated in to English by authors.

FGDs were carried out with all groups separately except SS and SKs who as CHWs were interviewed together. Focus group sizes ranged from 7 to 9, lasted about 1 to 2 hours and took place at the house of one of the participants to provide a familiar environment. The purpose of the FGDs was to obtain additional information on the issues identified in the KIIs. As standard sizes for FGD were not met, CHWs took part in an IGD. Also they were interviewed within their working hours at the office setting that was not fully supportive to conduct KII as well. All FGDs were conducted by trained RAs. The participants were encouraged to respond to all the issues raised by the facilitator. Researchers were alert to unexpected and new responses and were able to probe effectively to gather additional in depth data.

The purpose of the KIIs was to gather information about personal experience of the research topic from knowledgeable and informative persons - the mothers and family members. KIIs were conducted at mostly to the respondent’s home. The average duration of KIIs was just about 45 min.

### Interview guides

A total of three interview guides and one checklist were prepared based on literature reviews and finalized after pre-testing in *Korail* slum of Dhaka city where *Manoshi*, an urban MNCH programme has been operating since 2007. Among the CHWs, only SK was interviewed by IGD using written checklist. This interview guide and a written checklist of study related topics were used for KIIs, FGDs, IGDs and semi-structured interviews.

### Data analysis

Semi-structured interviews were analyzed using framework analysis and KIIs, FGDs and IGDs by content analysis [32, 33]. The interview transcripts were read repeatedly by the researchers, meaning units relevant to the research questions were highlighted and ‘in-vivo’ codes were also developed. A coding index was developed initially and was constantly refined throughout the data analysis when new insights emerged. Then categories were developed by considering each paragraph of the transcript in an attempt to summarise what respondents were saying in relation to our research questions. Emerging themes were developed from the categories, compared and modified with each and every independent transcript. Table 2 is a typical example how the data was analyzed. For example, the theme about the difficulty of conveying messages to women through EE was established by a process of four steps that includes identification of meaning unit, category, sub-category, and sub-theme. Different themes were incorporated under a broader theme such as *acceptability of EE*. In order to illustrate each theme, quotations were selected and presented in the results section based on their representativeness. To reduce the possibility of non-sampling error, to support the trustworthiness of information and to check and establish validity, triangulation across different interviews and discussions was done [34] Researchers regularly discussed interpretation of specific pieces of data; the robustness of the relationship between categories, sub-categories and sub-themes and between the themes identified and the research questions.

**Table 2:** Examples of matrix table of one theme from content analysis: difficulty to reach messages

Transcript/meaning unit	Sub-category/possible closest meaning	Category	Sub-theme	Theme
*All family members went there except 1 or 2 who had work in the home. The ‘Para’ (an area) was big so most of the women were there because it was near to their home* (ID: R)	Most of the women can watch street theatre if the setting is near to their household.	importance of settings and distance for street theatre	Barriers to watching street theatre	Difficulty in conveying messages to the women through entertainment education
*Women of this village watched the programme of local song. They said local songs were performed by BRAC. Apa (CHW) had asked women to join the event. I could not enjoy it because of household work.*(ID: R)	Women could not enjoy the entertainment due to work despite previous information about the event from CHW.	Traditional norms of household work are barriers to watching local performances	Barriers to watching local performances	

## Results

Major characteristics obtained from interviewing the participants were age, education, occupation and religion. The pregnant and lactating mothers had an age range of 15 to 35 years. Most had a primary education, few were illiterate and one had a bachelor degree. Most were housewives and few were service holders, day labourers, van drivers/cart driver (in local Bengali term it is called ‘*thelagari’*), food shop owners and teachers. The age range of male participants was 22 to 40 years, most of them were literate and few were illiterate. Apart from businessmen, there were also rickshaw pullers, village doctors (VD), farmers and day labourers. Mother-in-laws had an age range of 33 to 57 years. They were mostly housewives and illiterate. The majority of the respondents were Muslims.

### Common sources of MNCH information

The respondents were found to be fully cognizant of the BCC tools used by the BRAC IMNCS programme. Thirty KII and FGD respondents were familiar with the doorstep home visits and IPC with CHWs; with the flipcharts used by CHWs, with posters and stickers given to participants during IPCs and with periodical *jarigaan* (folk song) and street theatres. Respondents also spoke about the government produced entertainment television drama called “*Shukhi Paribar*” (approximate English equivalent ‘The happy family’) and the Maternal and Child Welfare Centre (MCWC) (in local dialect “*Shishu Mangal*”) as other common sources of information.

We report findings of community perceptions of BRAC’s BCC interventions first in the context of the research questions.

### Acceptance of and challenges inherent in different forms of media

#### Acceptance and challenges regarding IPC

IPC was appreciated by people because firstly, it involved the use of a common local dialect and mutual understanding of cultural issues that led to easy understanding of the messages. Secondly, IPC led to rapport building and consequently the ability and confidence on the part of community members to communicate directly with CHWs.

*“As we can ask questions without any hesitation, they speak like us. I do not have any problems in recognizing their language*”. (SSI, Pregnant women)

However, respondents also expressed concerns about CHWs lower level of education, training, limited skill in detecting health problems and low social status. Therefore, some felt that they could not depend on them for pregnancy management and found their services unacceptable.

*“Rich and higher educated people (in the village) think that CHWs do not know anything. They do not have faith in their training. So they ignored them”*. (KII, Husband of a pregnant woman)

Several respondents urged that CHWs should have scheduled meetings with the community in group settings. One common demand was for the presence of higher officials in the meetings, which would bring more credibility to local workers. A typical comments was,

*“The group meeting should take place once in a month and will be better if higher officers come and conduct these meetings other than Shebika (SS) or Kormi (SK)”.* (KII, Husband of a pregnant woman)

Many participants recommended increasing the number of home visits by CHWs to help in repetition and memorizing the messages and to place more emphasison visits for the neonates and children under-5. Mostly the female respondents mentioned,

*“If the Shasthaya Kormiapa (SK) comes frequently and makes us understand then we will memorize more. They come once in a month. That is why we could not memorize those (messages)”.* (FGD, Pregnant woman)

#### Acceptance and challenges regarding printed materials

All of the participants mentioned that they have seen and received all the posters and stickers. It was found that, among all the posters, the one with a smiling and healthy looking mother and a child was accepted by most. The majority of respondents have seen this and liked to display this poster in their home. Respondents were also eager to collect multiple copies of this poster.

There is a symbol of a mobile phone at the bottom of each poster and sticker with a space to write a phone number of either the CHW or a BRAC staff member to be contacted in case of emergency. According to the respondents, the pictures and messages are helpful in alerting them to danger signs and early actions to be taken.

*“They (CHWs) came and gave these,…I glued it in my home. Whoever comes can see these and they can be noticed all the time, which will help us to be alert. If any problem occurs, she can be taken to the hospital. By seeing these pictures we can memorize these well”.* (KII, Mother-in-law)

Positive feedback was also from husbands regarding the printed materials.

“*When my wife started bleeding during her pregnancy, I instantly recognized it as a danger because I had seen the picture of danger signs before and Shebika apa (SS) gave the message too*” (FGD, Husband of a pregnant woman).

However, some associated the posters about danger with notions of ‘bad fate’ for both mother and child.

*“We do not have money. How do we feel good incase of such danger?*

*I am scared of these if it happens to me. From where and how can we manage money? How will we be saved?”* (KII, Pregnant woman).

Regarding issues of display, some of the pregnant and lactating mothers felt that it would be a shameful thing to display pictures of maternal danger signs inside their room from where they could be seen by the male relatives and parents-in-law. So they were glued either at the corner of the room, on the back of the door or even inside drawers, where they were not immediately obvious. According to CHWs, elderly people did not like open displays of stickers inside the room as some community members believe that prayer (*namaj*) is impossible with illustrations of human beings hung inside the room. In some cases, children of the household also removed the posters from the door.

#### Acceptance and challenges regarding EE

The majority of the respondents were aware of folk song and street theatre but few had the opportunity to actually listen to or watch these. According to them, children and women were the main audience. It was found that folk songs attracted people’s attention very quickly. Songs were a means of disseminating multiple messages in a short time and in an amusing way. Most of the female respondents reported that during CHW visits at home usually their children, household head and other family members were absent. Therefore, all the family members could enjoy and learn from these events taking place in open community settings like market places.

*“…..different types of people including young, adult, married and unmarried people, like folksong. Shebika (SS) visits and talks only with the pregnant mothers. But folksong is understandable to all, whether men or women”.* (KII, Pregnant woman)

Street theatres were valued by many because the dramatic enactment of messages supplemented the use of song and speaking alone.

*“Drama is performed in front of us. It is understandable. It is performed so nicely. Many people come and watch together and if I forget any point of the drama, other people can help me to remember. That is why I like drama more than other media”.* (KII, Mother of neonate).

Pregnant and lactating women shared common obstacles with mother-in-laws regarding EE these were distance, household chores, inappropriate timing and lack of prior notice. They also faced other barriers like the need to obtain their husband’s consent and lack of social acceptance for attending what was, essentially, a public event. Occupational work-loads and lack of interest were the barriers for men.

Community people suggested a change in timing and wanted these events more frequently. FGD participants recommended that these events take place fortnightly. Also, it would have been easier for the women to access if EE was arranged nearer to their homes, or if adequate prior notice was given.

#### Acceptance and challenges regarding mass media

A variety of health related television programmes on polio vaccination, diet of pregnant women and family planning were also enjoyed by the women. Among them, ‘*Shukhi paribar’* was the most viewed and enjoyed programme, with rich information on MNCH as well as general health. Generally, female household members, neighbours and children enjoyed the programme together. The general viewers felt that the stories were clearly based on experiences of rural communities, that the actors and actresses spoke with rural accents and their movements, attire and attitude were similar to those of the viewers.

*“I like this programme because the programme represents our words (views). I can relate the situation with my real life by watching this programme”*. (SSI, Pregnant woman)

The barriers to viewing these TV programmes were for women household work and child care and for men, lack of interest and workload. A pregnant mother said,

*“What will we discuss (about the shukhi paribar)? He does not like this programme, rather, he likes hindi movies. He has no interest in this programme. That is why I do not tell him anything about it*”. (KII, Pregnant woman)

### (b): Comprehensibility of terms, illustrated materials and audio-visuals

The use of local dialect by the CHWs helped community members to easily understand the MNCH related terms such as antenatal care (*gorvobotirjotno*), diet (*gorvobotir khabar*), heavy work (*varikaaj*), personal hygiene (*poriskar porichhonnota*), birth preparedness (*purbo prostuti*) for pregnant women, TT vaccination (*sui deya* lit. giving the needle), supplementary food for children (*shisur barti khabar*) and maternal and newborn danger signs (*maa o shisur bipod-chinho*).

Participants could understand the benefits of the messages and clearly describe most of the pictures except for a few, such as the five maternal danger signs, retained placenta and presence of a skilled birth attendant. Because of the lack of comprehensible visual cues, participants could not differentiate whether the illustrated problem occurred before, during or after delivery. The picture showing headache and blurred vision of eclampsia was confused with a woman feeling tired. People thought the pictures related to colostrums feeding and retained placenta were the same. The illustrations also failed to highlight the need to make emergency calls in these dangerous situations. The illustration of a skilled birth attendant assisting at delivery was mistaken for the mother-in-law or mother. However, as mentioned above, the illustrated messages became clear when accompanied by explanations from CHWs.

“*Apa gave the sticker (maternal danger signs), showed the pictures to me and my mother-in-law and pointed out specific pictures of bleeding, prolonged labor and convulsion…I know that if more than twelve hours have passed then it is called prolonged labor. You need to be taken to the hospital.”* (KII, Pregnant woman)

Respondents considered that frequent visits of the CHW and watching of theatre performances or hearing the folk songs can be useful for memorizing messages. Most of the respondents had the ability to recall the messages of a drama. It was found that visualization helped pregnant and lactating women to understand the messages given by CHWs. For example, they accurately recounted messages about ensuring pregnant women taking extra food, rest and proper sleep during pregnancy and about colostrum feeding. As with the SK’s use of flipcharts, both EE and TV also have the advantage of using both audio and visual techniques resulting in better story recall and message interpretation.

“*I like the part of the drama when the husband of a pregnant woman took her to the hospital in a van during a complication.”* (FGD, Husband of a pregnant woman)

### (c): Reported influence of BCC messages

People were sensitized about maternal and newborn care especially the danger signs. Conversations occurred between husband and wife regarding MNCH issues. Mutual understandings were influential in decision making regarding family planning, the use of health services and child rearing.

*“Let us drop the mistakes which we have done before, mistakes which have occurred during our marriage, just let go. We watched, heard and whatever we learnt we will implement. We will not repeat the same mistakes.”* (KII, Husband of a pregnant woman after watching drama)

Sharing of MNCH messages had increased among peer groups, relatives, neighbours and the elderly. According to the CHWs, this made pregnancy identification easier for them than before. Participants could easily recall MNCH messages and considered themselves to be more conscious in taking the right health promoting decisions for their own wellbeing and development.

“*Now we keep ourselves clean and take good care of our baby which we did not do before. Earlier we did not maintain any health rules that we do now and it makes our lives healthy and better*”. (KII, Mother of an under-5 year old child)

## Discussion

### Summary of findings

This study aimed to explore community perceptions of the BCC intervention of the BRAC IMNCS programme in rural Bangladesh. IPC channels were found to influence not only community’s knowledge, attitudes and motivation but also effect reported behaviour change. Mass media like drama, folk song (*jarigaan)* and TV were also reported as important and credible sources of information. However, challenges identified included perceptions of CHWs’ low educational and social status; difficulty in understanding and limited cultural acceptance of some illustrations particularly the maternal and newborn danger signs; inappropriate timing and location of folk song and street theatre performances and limited coverage of secondary audiences like men and the elderly.

### Importance of interpersonal communication

The study showed that in the community, IPC was very important. IPC facilitated the exchange information, importantly in ‘our words’, expression of feelings and the receipt of immediate feedback. This meant that doubts and misconceptions could be immediately dealt with and ongoing mutual support developed between health workers and community members and between community members themselves so that new knowledge and behaviours were reinforced. Furthermore, in case of emergency, community members were confident to contact BRAC staff directly by phone. This finding is consistent with another study on changing community attitudes [35, 36]. Although our study did not compare the effectiveness of IPC with EE, Hussein et al. reported that IPC was more effective than other media when the messages were targeted to lay people [37, 38]. Two way interactions are deemed essential for identifying the level of readiness for change and to support and convince individuals to adopt health-prompting behaviour [39]. While messages were being communicated, we found no evidence from community members or CHWS that CHWs are specifically assessing individual’s level of readiness to change.

BRAC has chosen the multidimensional approach to BCC. The context in Bangladesh is one of rapid *social* and economic development predicated on community mobilization and empowerment, especially of women. In this context, it is likely that the theoretical need to assess *individual’s* level of readiness to change is less important than working with communities, both men and women and across generations for change as the BRAC IMNCS BCC programme already does [40, 41]. The CHWs are key in communicating and chosen as peer educators because they belong to the same community and have similar socio-cultural backgrounds and face challenges akin to those of the programme participants. The female respondents also share feelings of fellowship with them based on a gender perspective. BCC theories, mostly social learning and diffusion of innovations theory, assert that credible peers can influence health behaviour change [42, 43].

A recent study on peer educators in HIV/AIDS prevention programmes revealed their positive contribution to prevent HIV/AIDS among adolescents [44]. However, CHWs are often illiterate or less educated, especially in our study area. Thus while on one hand, CHWs are easily understood and have a growing credibility, on the other hand their lack of formal education, an important status marker in Bangladesh, was considered as a disadvantage in communication. At programme level, CHWs’ technical and communication performance has an impact on their growing credibility therefore the programme should replace refresher training with regular in-service training that is rooted in an experience-based problem solving approach. In this way CHWs ability to assess, manage and take right decisions in saving mothers’, newborns’ and children’s lives will be incrementally increased. In addition, because time spent during IPC is important for increased rapport-building and negotiation about actions to be taken by the family, the technical medical skills in the CHW training programme should be strongly buttressed by further regular training in assertive two way communication [45]. In the interim, CHWs’ credibility can be enhanced by having their managers and senior officers formally introduce them and by the presence of higher officials in certain group meetings. In the longer term, another way might be for BRAC and other NGO CHWs training and practice to be formally accredited through government and related technical body affiliations. However, since the initial development of Primary Health Care in 1978, (WHO) formal accreditation of CHWs has always been strongly contested because of the need for both sustained political will and long term additional resource [46–48].

### Printed materials

We have reported on the research participants’ functional literacy. We also reported community members’ concern to memorise messages. This derives from the importance of recall in a rural community that, barely three generations ago, depended almost entirely on oral communication with a literacy rate of just 16.8 % in 1971 [49]. Also, school education in Bangladesh, both religious and secular, traditionally includes a lot of repetition and memorization. It is not unexpected therefore, that community members wish to memorise the messages. However, it is important that memorization is not the aim of BCC but rather that memorization prompts timely, appropriate action. Our printed materials included both Bengali text and illustrations and were produced with the objective of being both an aid to IPC and a stand-alone communication medium with this rural population.

The concept of visual literacy refers to the capacity of persons to code and decode visual signs other than words. Visual literacy was first discussed in relation to health promotion by Fuglesangin 1973 [50]. Although pictures are considered as an effective way to communicate with the rural illiterate people, we found two types of barrier to their comprehension of BRAC MNCH printed materials. The first is the fact that although there was some pretesting of the BRAC materials, some of our illustrations were ineffective as ‘stand alone’ communication media, for example the picture of the pre-eclamptic woman with a headache was initially interpreted by our participants as being a ‘tired woman.’ In the interim, misconceptions regarding some of the existing illustrations can be corrected by reinforcing the training of CHWs to assist in materials comprehension through IPC. In the medium term, the solution to this problem is in the rigorous and repeated pretesting of illustrations with the target population prior to printing new editions. Pre-test and adaptation of materials needs to continue until the community members’ answer to the question, ‘What do you see here?’ aligns with the intention of the designer and health communicator [51]. The cost of pretesting can be offset against that of the time used by CHWs to explain poorly designed materials. Nonetheless, the programme should not ignore the importance of IPC aided by the flipchart in creating a sympathetic connection between CHWs and community members [52] and the ongoing usefulness of the stickers as a resource that is available to the families and wider community in the absence of CHWs.

Our rural respondents successfully used culturally acceptable ways of displaying and retaining posters and stickers keeping them in drawers or using rice or flour paste to stick communication materials to smooth mud walls and behind wooden doors. This contrasts sharply with the challenges faced by Dhaka slum dwellers in the sister Manoshi programme [7]. Living in rented accommodation, some landlords objected to the women displaying these materials and tore them down. Nor did landlords allow nails to be used for fixing posters. Also, urban respondents deemed posters unattractive when stuck to walls made of corrugated iron and other irregular surfaced, often recycled building material. It might be that these female urban slum dwellers, whose 22 % literacy rate [7] compares unfavourably with literacy of female respondents in Nilphamari (53 %) in 2010 [23] and who have less access to public space than their rural sisters, are also less able to interpret illustrations and particularly those posted on uneven surfaces which requires the ability to ‘read’ perspective.

Secondly, religious and cultural barriers were found in accepting pictures like those in the BRAC stickers of maternal and newborn danger signs. This barrier derived from the participants concern about developing the same ‘bad fate’ as the complication shown in the illustrations. The ‘bad fate’ barrier however operated less in the context of cultural conservatism and more in the context of the fear of not being able to get treatment because of their poverty and the cost involved in using health services. BRAC already uses birth preparedness messages including saving during the antenatal period for possible medical emergencies. Birth preparedness messages therefore need to be reinforced and more closely aligned to the messages about danger signs and immediate telephone contact with BRAC staff, (already a source of security and assurance), once danger signs are recognised.

At a societal level, community members find themselves in what Van Gennep [53] described as a ‘liminal’ state. It is not surprising therefore that our respondents were able to hold seemingly conflicting notions of bad fate and fear of economic cost closely together. This response resonates with Lambert’s analysis of the way Rajasthan is used ‘bad fate’ to exert agency and manage their illnesses [54]. More recent work [55] about client and health worker satisfaction with inpatient delivery care in northern Bangladesh found that respondents gave a similarly sophisticated and nuanced analysis of what make them, at one and the same time, satisfied and dissatisfied. Both users and providers described in some detail the limitations of the current but constantly changing and developing service. Like our respondents, they are living in a liminal state.

Although BRAC’s local MNCH committees are supposed to give voice to the concerns of local people about health services, the functioning of the committee was also hampered by complex tensions between committee members, especially where those with lower social status had higher formal education level than acknowledged community leaders. However, by using mechanisms that are similar to traditional patron-client relations, MNCH committee members have had modest success in motivating and encouraging community members to follow practices recommended for improved MNCH. There were some reports of improved timekeeping and behaviour with patients by health facility staff as a result of follow-up by MNCH committee members [56]. The IMNCS programme therefore needs to work with acknowledged community leaders to improve their technical knowledge of MNCH. In the context of growing gender equality, this should become easier as the distance traditional high status male leaders keep from dirty (*napak)* ‘women’s matters’ is reduced.

At a macro level, BRAC’s Health and Human Rights and Legal Aid Service (HRLAS) senior staff advocate health services having a legal ‘duty of care’. However, the enactment of such legislation is politically contentious, given the power of the medical establishment. HRLAS could also develop specific rights based messages about negotiating hospital admission, treatment and cost. It could also, potentially, extend it’s legal services to those who, in response to BCC, have tried to use secondary level health services and received inadequate care. A common persistent problem is that of untrained or absent ‘consultant’ obstetricians and anaesthetists [57]. This problem is a function of increasing utilization of health services, inappropriate staffing configurations, and weak administration of vacant posts and unauthorised absences [57, 58].

At a national level, Government and NGOs need to address the financial barrier by improving health service coverage and sustaining recent interventions such as health insurance or community based financing for rural people, especially the poor. Proper advertisement, careful supervision and monitoring of any such attempt should be present to ensure such investments reach the rural poor.

### Education entertainment

Although messages through EE and mass media had limited reach to the female members of the community, women who did attend EE could easily relate the situations played out with their own lives and perceived realities. BCC through drama and songs was also a very popular mode of communication reported in other studies [13]. Research also confirmed EE and mass media as highly acceptable and effective for audiences with limited formal education [59–62]. Keeping community people’s eagerness for EE in mind, low cost and low tech communications like local traditional folk songs and theatres may be effective for promoting social and behavioural change. Careful organization and advertising are also important in addition to holding these events. Also, community television with big gatherings or the use of tablets with smaller gatherings and posting videos of *Jarigan* and street theatre on You Tube can be another way to broadcast appealing health related programmes. The use of tablet computers is already being piloted in by Plan International in Nilphamari [63] and by some government community clinics and health workers [64].

Overall, our research revealed there was less coverage of secondary audiences, particularly men. In the light of the importance of target segmentation in BCC [65] it is important that the programme should research, design and test more effective BCC methods such as focused meetings; flash cards including the role of men in pregnancy, delivery and postpartum care; and delivery preparedness to increase men’s comprehension and participation in these issues.

This study was not designed to demonstrate behaviour change (before and after) the BRAC MNCH BCC intervention. Our findings about community perceptions of the BCC tools, reveal that a well co-ordinated, simultaneous and repeated use of different channels are likely to continue to be useful to communicate and reinforce key, carefully structured messages and support changing behaviours throughout the community, including men and older persons. In line with other communication research findings, interpersonal communication between CHWs and community members at home visits and group meetings remain immensely important. However, the BRAC IMNCS BCC programme can be further enhanced by the development of gendered storylines and attention to the seasonal cycles of conception, birth, postnatal care and childhood illness with broadcast of seasonally relevant messages. We recommend that messages and media be rigorously pretested before going to scale. While live street theatre and folk song (*Jarigan*) are well accepted, their usefulness could be expanded by uploading videos of these programmes to social media. Similarly print materials could be uploaded to the web. To increase synchronicity of messaging, mass texting to both CHWs and community members could be used to increase coverage and sustainability. Future research should identify how best to combine IPC, printed materials, traditional cultural forms, social media and mass media in different field situations.

### Limitations and methodological considerations

One of the limitations of this qualitative study is that it did not measure the contribution of each BCC component in influencing behaviour change. This was not the intent of this study. Furthermore, health promotion and behaviour change communication researchers are cautious about their ability to disaggregate the impact of different communication media in a multi component intervention [66, 67]. More recently, although some studies discuss the use of randomised control trials in behavioural interventions they conclude that ‘When interventions are complex, pragmatic trials may be more likely to succeed that explanatory ones’ [68, 69]. In addition, the IMNCS was operating in the context of a long history of successful community based interventions that date back to the promotion of the use of oral rehydration solution in the late 1970s [70] and the need to inform the programme in the light of the rapidly approaching target date for the Millenium Development Goals.

As a BRAC researcher, some bias may be present by conducting the research in the BRAC intervention area. Using convenience sampling in selecting the study area is another limitation because some research participants might be missed in remote areas. In qualitative research, there are also possibilities of misinterpretation, loss of information and biases due to translators’ interpretation and assumption [71–73]. Triangulation of information from different groups such as pregnant and postpartum women; husbands of pregnant and postpartum women; mothers and fathers of newborns and under-5 children; mother-in-laws and CHWs using FGD, KII and IGD was a useful strategy for checking consistency and also disagreement within and across the groups [34].

## Conclusions

The study provides in-depth information about community members’ awareness of and regard for BRAC MNCH BCC; what they want from it and what BCC should be like in the future. We have revealed how interpersonal communication is important; how with more rigorous pretesting, existing printed materials can be improved and suggest that the programme can experiment with the as yet unused social media and text messaging to increase the coverage of BCC in the community and to alert CHWs to seasonally relevant messages.. Because we are looking to effect ongoing behaviour change, research should continue to understand in a more nuanced way the community’s changing perceptions regarding BCC, the barriers to behaviour change; the opportunities for improved integration and widening coverage of messaging, together with building community support for new behaviours leading to improved health outcomes for mothers, newborns and children.

## Additional files

Additional file 1: Key informant interview guide. (PDF 16 kb) Additional file 2: Informal group discussion guide. (PDF 21 kb)

## Abbreviation

ANC: Antenatal Care
BCC: Behaviour Change Communication
CHW: Community Health Worker
DOTS: Daily Observed Treatment, Short Course
EE: Entertainment Education
FGD: Focus Group Discussion
HRLAS: Health and Human Rights and Legal Aid Service
IGD: Informal Group Discussion
IMNCS: Improving Maternal Neonatal and Child Survival
IPC: Interpersonal Communication
KII: Key Informant Interview
MCWC: Maternal Child Welfare Centre
MNCH: Maternal Neonatal and Child Heath
PNC: Post-Natal Care
PO: Programme Organizer
RA: Research Assistant
RED: Research and Evaluation Division
SK: Shasthya Karmi
SS: Shasthya Shebika
TB: Tuberculosis
TT: Tetanus Toxoid
