# Supplementary material for: Community perceptions of behaviour change communication interventions of the maternal neonatal and child health programme in rural Bangladesh: an exploratory study
Source: BMC Health Serv Res. 2016 Aug 16;16:389. doi: 10.1186/s12913-016-1632-y (PMC4987986; doi:10.1186/s12913-016-1632-y)
Supplement: Additional file 1: — Key informant interview guide. (PDF 16 kb) [file 12913_2016_1632_MOESM1_ESM.pdf]

Key informant interview guide

**Name (Optional):**	**Sex:**
**Place of interview:**	**Age:**
**Date of interview:**	**Main occupation:**
**Interviewer name**	**Education:**
**Time interview started:**	**Time interview ended:**

The following questions is only for the pregnant women and mother of neonate and under five years of children

1: **Awareness of BCC media and messages**

- What do you know about BRAC MNCH programme? (If yes) From whom did you know?
- Do you know about BRAC SS/SK? Does she visit in your home? How frequently? How many times did she visited last 1 month? What they do? What she/they have told? How?
- How she /they delivered the messages?(verbally, with the help of sticker, poster, flipchart)
- Did you ever see the poster/sticker/flipchart? (If yes) where did you see those? Who provides? When? Why?

2 **Acceptability (To what extent are BCC media attractive to the community members)**

- What is your comments/views about community health workers visit, communication with you, folk song and street theater (ask one by one specifically)
- Did/Do you share the information to others/family? How do your family members react?
- Did you keep all posters in your home? What about you/families perception regarding poster etc.

2: **Message comprehensibility**

- Do you understand all those messages that SS/SK told? (if verbally) Do you understand all the terms, words, phases etc.
- What were the contents of poster/sticker/flipchart?
- Which picture you likes/dislikes more? Why
- Have you ever heard or seen any folk song or drama regarding maternal, neonatal

and child health arranged by BRAC? (If Yes) Where? When? How many times? Who performed? How long ago? What was the content? (If No) What was the reason? (Ask about timing of performance and other problems?) Liking or disliking about the folk song/ drama (Which part he/she likes/dislikes more? Why).

- Please share best part of interpersonal communication, folk song and street theater. Why do you think it is best? How?

3: **Personal Experience (Are these messages able to influence community trust)**

- Can you relate any of those messages with real life? Please give some example. (Elaborate)
- How to make all those messages more communicative?
- Do you think there is any change needed at pictorial materials? (If Yes) Which one and why? What would be the possible changes?

<u>The following questions is only for Father and mother in Law</u>

- What do you think why SS/SK come to your household? What they say to you/your wife/daughter in law?
- Are they gave any posters/stickers? What about that? Why they provide you posters/ stickers? Why they show you/your wife/your daughter in law all the pictures?
- What problems do you usually face when CHWs deliver health messages using BCC tools? Why /why not?
- Have you ever seen folk song/drama arranged by BRAC? What about that? What are the purposes of folk song or drama?
- What is your view/opinion about different BCC tools? (Ask about problems/which messages they followed willingly/Which they showed rigidness)
- What do you think, which BCC tool is more effective and communicative? (Mention all BCC tools (Posters, stickers, flip chart, folk songs, drama, verbal message). How?
- Please share best part of interpersonal communication, folk song and street theater. Why do you think it is best? How?
- Can you relate any of those messages with real life? Please give some example. (Elaborate).

Closing Questions

- Do you feel there is something important we should have asked that we did not address?

[Thank you very much for your time]
