# Supplementary material for: Community perceptions of behaviour change communication interventions of the maternal neonatal and child health programme in rural Bangladesh: an exploratory study
Source: BMC Health Serv Res. 2016 Aug 16;16:389. doi: 10.1186/s12913-016-1632-y (PMC4987986; doi:10.1186/s12913-016-1632-y)
Supplement: Additional file 2: — Informal group discussion guide. (PDF 21 kb) [file 12913_2016_1632_MOESM2_ESM.pdf]

Informal group discussion guide

Profession :	Name: (Optional)
Place of interview:	Sex:
Date of interview:	Interviewer name:
Time interview started:	Time interview ended:

General background:

- Please tell us about your work
- How long have you lived in this community?

What extent are BCC media attractive to the community members?

- What problems do you usually face in delivering health messages using BCC tools? (Why /why not?)
- What is your view/opinion about community perception on different BCC tools? (Ask about problems/which messages they followed willingly/Which they showed rigidness)
- What do you think, which BCC tool is more effective and communicative? (Mention all BCC tools (Posters, stickers, flip chart, folk songs, drama, verbal message)

Are these messages able to influence community practice// Do the messages appeal to the beliefs of the target audience

- Can you share your experience that focused how community people are benefitted with the type of strategies?
- What do you think how community people are influenced from these messages.

Recommendation

- How to make all those messages more communicative?
- Based on your experience, do you think there is any change needed at pictorial materials? (If Yes) Which one and why? What would be the possible changes?

[Thank you very much for your time]
